# La Grippe

**Published:** 1890-07

**Authors:** 

**Affiliations:** Kiel. A. H. Z.


### LA GRIPPE.

(Some Thoughts on the Late Influenza, by Dr. Kunkel, of Kiel. A. H. Z., 11, 90.)

Perhaps many a person suffering from la grippe never asked for medical advice and recovered anyhow, but children were often, from the very start, severely handled. Headache and vomiting all through the attack were witnessed in most patients, and Aconite, given from the start, seemed to cut short the attack. Cough was the most troublesome symptom; *Sabadilla* seemed indicated for a dry cough, worse before midnight and during east wind; *Arsenicum*, worse after midnight, and children had to sit up when severe coughing set in; sleeplessness, nightmare, waking up frightened, deliria, thirst, but drinking aggravates the cough. *Veratrum-album*, aggravation before midnight; great thirst and desire for large quantities of cold drink, but worse after drinking; general coldness of body, great malaise, cold frontal sweat. *Phosphorus*, laryngeal cough with sensitiveness of the larynx, hoarseness, pleuritic or bronchitic symptoms, palpitations, pains around heart, sweat during sleep, which disappears when waking up, must sit up for relief; vertigo, sleepy in daytime, especially afternoons. *Cuprum*, a worrying cough at different times of day in children, with spasms, turning eyes upward, bluish tint of face, twitchings during sleep. *Bryonia*, cough with pleuritic manifestations, etc. *Carbo-veg.* 3 or 6, cough with hoarseness, burning pain in larynx, worse before midnight, gastric symptoms heavily coated tongue, foul breath, flatulency.

The grippe becomes dangerous by its sequelæ, especially pneumonia, whose appearance had nothing in common with genuine pneumonia, no initial chill, etc., spreading rather in a sneaking manner, from the pleura or bronchi, and when from the former, Bryonia often sufficed for a cure. Broncho-pneumonia was often preceded for several days by cough of a spasmodic character. Gradually short breathing set in and here Arsenicum acted well, also Phosphorus, according to their indication, and Sulphur had to be interpolated where psora showed its cloven foot. Sometimes the indications could not be relied on, as the picture of the disease was constantly changing and the physician felt grateful when the cough ceased, though his intervention could not be praised for it. *Cuprum* was our stand-by in the advanced stages of complicating pneumonia, for cardiac paralysis often suddenly finishes the case. Sudden attacks of suffocation with deadly anguish set in, the diaphragm is in continuous action, while the thoracic muscles seemed to have ceased to labor, and if Cuprum 3d, 6th, or 30th fail to prevent a second attack, the patient's doom is clearly foreshadowed.

I treated an old lady of seventy years, who, after a severe attack of influenza was taken down with right-sided pneumonia, and though Bryonia and Phosphorus removed the inflammation, collapse set in with total aphonia from mere weakness, pulse small and irregular. Cuprum saved her life. About a week ago I had to visit a little girl of four years, whose sister recovered from diphtheria under Mercur-cyanatus, but it failed in her case, and Aurum was substituted, which ameliorated somewhat, but it invaded the larynx with dyspnœa, hoarseness, a thickly coated, yellowish-brown tongue, especially at the root (a keynote for Carbo-veg.), foul breath. In the evening I prescribed Carbo-veg. 3d c., the medicine acted so well that dyspnœa and hoarseness disappeared, the child fell into a quiet sleep, but died suddenly in the morning. Cuprum might have saved this life. In fact, the most grave case of diphtheria may yield most beautifully to our treatment, and still they do not exclude the danger of a final cardiac paralysis, when everything looks favorable for a speedy recovery.

In several cases of diphtheritis, otitis media suppurativa was a disagreeable complication, but yielding to Silicea and Calcarea-carb. In a little babe of eight weeks, meningitis with its cri-hydrocephalique followed. Apis 3d c., was given successfully, but the suppuration lasted longer, sometimes interrupted by vomiting. Silicea and Calcarea in repeated doses finally removed this psoric complication.

Influenza, in fact, is worse than cholera, for it causes recrudescence of old sufferings, and it is well for the patient when we can find out what remedies suited his individual constitution and helped him before. Anamnesis first and the remedy is more easily found.

